# Astragaloside IV mitigates influenza-induced inflammatory responses by suppressing the Wnt/β-catenin signalling pathway in alveolar macrophages

**DOI:** 10.1186/s13567-025-01529-5

**Published:** 2025-04-30

**Authors:** Jianli Tang, Yu Gao, Yuchen Fu, Zhaoqing Han, Ping Xu, Xin Li, Shuaiyong Wang, Xin Wang

**Affiliations:** 1 College of Agriculture and Forestry Science, Linyi University, Linyi, 276000 Shandong China; 2 College of Animal Science and Technology, Shandong Agricultural University, Taian, 271018 China; 3 Carter Immunology Center, University of Virginia, Charlottesville, VA USA

**Keywords:** Astragaloside IV, alveolar macrophages, respiratory virus, inflammatory response, Wnt/β-catenin signalling pathway

## Abstract

Respiratory viruses, including the influenza virus (IAV) and severe acute respiratory syndrome coronavirus 2 (SARS-CoV-2), pose significant health threats. As tissue-resident macrophages, alveolar macrophages (AM) are crucial for defending against respiratory viral infection by producing cytokines, engulfing virus-infected cells, and promoting wound healing. However, excessive inflammatory responses can lead to tissue injury. Growing evidence indicates that astragaloside IV (AST IV) regulates innate immune responses. Specifically, AST IV balances the inflammatory response to mitigate tissue damage and promote tissue repair. However, whether AST IV directly targets AM to alleviate lung damage induced by respiratory viral infection remains unclear. Our results demonstrate that AST IV treatment significantly reduces morbidity and mortality in mice during IAV infection. AST IV markedly decreases proinflammatory cytokine levels, mitigates lung injury and promotes lung recovery through enhancing the repair capacity mediated by alveolar type II cells. Mechanistically, AST IV suppresses the Wnt/β-catenin signalling pathway, which is critical for driving inflammatory responses in AM while maintaining mitochondrial fitness. Thus, our findings suggest that AST IV effectively targets AM to alleviate inflammation and lung damage caused by respiratory viral infections, highlighting its potential as a therapeutic agent for managing viral pneumonia.

**Supplementary Information:**

The online version contains supplementary material available at 10.1186/s13567-025-01529-5.

## Introduction

Although numerous effective vaccines and drugs have been developed, respiratory viruses such as influenza A virus (IAV) and severe acute respiratory syndrome coronavirus 2 (SARS-CoV-2) remain significant public health challenges, primarily due to the ongoing emergence of new viral variants [1, 2]. Therefore, a comprehensive understanding of the complex molecular mechanisms underlying respiratory virus pathogenesis is crucial for developing broad-spectrum therapeutic strategies.

As tissue-resident macrophages, alveolar macrophages (AM) are essential for lung immunity and homeostasis. Upon pathogen invasion, AM protect against pathogens by recognizing specific receptors, engulfing and eliminating them through phagocytosis, and secreting cytokines and chemokines to recruit and activate other immune cells [3, 4]. Additionally, AM promote tissue repair and resolve inflammation, making them essential for lung defense and recovery [5, 6]. Importantly, excessive inflammatory responses can damage the tissue, highlighting the importance of maintaining a balanced inflammatory reaction. Given the dual role of AM in regulating inflammatory responses, targeting AM represent a promising therapeutic strategy for treating respiratory viral infections.

Astragaloside IV (AST IV) has attracted considerable attention for its potent immunomodulatory properties, especially in alleviating excessive inflammatory responses and fibrosis triggered by various stimuli. AST IV mitigates heatstroke-induced brain injury and neuroinflammation in male mice by modulating microglial polarization through the PI3K/Akt signalling pathway [7]. In chronic obstructive pulmonary disease patients (COPD), AST IV has protective effects on lung function and anti-pulmonary fibrosis activity by inhibiting the GTP‒GDP domain of RAS, thereby downregulating the RAS/RAF/FoxO signalling pathway [8]. Furthermore, AST IV abrogates oxidative stress, inflammation, and EMT, effectively alleviating diabetic nephropathy and protecting against high glucose-induced renal injury [9]. However, whether AST IV targets AM to regulate inflammatory responses and the specific molecular mechanisms involved remain unclear. Furthermore, AST IV is involved in regulating influenza virus infection. For example, AST IV suppresses H1N1-induced inflammation through enhancing autophagy [10]. Moreover, AST IV alleviates the inflammation caused by influenza virus through targeting the NOD-like receptor thermal protein domain associated protein 3 (NLRP3)/Caspase-1 signalling pathway [11]. Thus, the multiple roles and details of the mechanisms through which AST IV modulates influenza pathology require further investigation.

The Wnt/β-catenin signalling pathway is a fundamental cell signalling pathway that governs diverse biological processes, including embryonic development, maintenance of tissue homeostasis, and regulation of cellular proliferation [12, 13]. In the absence of Wnt ligands, β-catenin is phosphorylated and degraded by a destruction complex. When Wnt ligands bind to their receptors, the destruction complex is inhibited, allowing β-catenin to stabilize, accumulate in the cytoplasm, and translocate to the nucleus, where it interacts with T-cell factor/lymphoid enhancer factor (TCF/LEF) transcription factors to regulate the expression of Wnt target genes [14]. Recent studies have highlighted significant crosstalk between the Wnt/β-catenin signalling pathway and inflammatory pathways, emphasizing its dual role in promoting or inhibiting inflammation depending on the context [15, 16]. β-catenin can modulate the activity of proinflammatory transcription factors such as NF-κB, thereby influencing the expression of cytokines, chemokines, and inflammatory mediators [15]. A recent study revealed that activation of β-catenin suppresses AM proliferation and stemness while promoting inflammatory activity, thereby exacerbating acute host morbidity and mortality during IAV and SARS-CoV-2 infection [17]. Accumulating findings suggest that AST IV is involved in regulating Wnt/β-catenin signalling. AST IV functions as a β-catenin inhibitor, suppressing the Wnt/β-catenin pathway to mitigate podocyte injury [18]. Conversely, AST IV enhances cognitive function and promotes hippocampal neurogenesis in stroke mice by downregulating interleukin-17 expression and activating the Wnt/β-catenin pathway [19]. Thus, further investigations are needed to explore whether AST IV regulates the Wnt/β-catenin pathway in AM to combat respiratory virus-induced excessive inflammation.

Here, we investigated the ability of AST IV to modulate the inflammatory response in AM and explored the underlying mechanism involved. Our findings revealed that AST IV reduces proinflammatory cytokines, mitigates lung injury, and enhances lung recovery by inhibiting the Wnt/β-catenin pathway and maintaining mitochondrial fitness. Importantly, we found that AST IV could serve as a promising therapeutic approach for treating respiratory viral infections.

## Materials and methods

### Ethics statement

The experiments were conducted using adult mice that were both sex- and age-matched. All animal procedures were conducted in strict accordance with the Animal Ethics Procedures and Guidelines of the Animal Ethics Committee of Linyi University (Number: LYUAEC 2024-000065).

### MH-S cell culture and drug treatment

The MH-S cell line (CellBank, Shanghai) was cultured in RPMI 1640 medium supplemented with 10% foetal bovine serum (FBS) and 1% penicillin‒streptomycin. The cells were maintained at 37 °C in a humidified atmosphere containing 5% CO_2_. MH-S cells were incubated overnight with astragalosides I–IV (MCE, NJ). Following incubation, the cells were harvested for cytotoxicity assessment and measurement of cytokine levels.

### Mouse viral infection and drug treatment

C57BL/6 mice were purchased from SPF Biotechnology Co., Ltd. (Beijing, China). Body weight and food intake were monitored weekly. Anaesthetized mice were intranasally inoculated with 200 PFU/mouse (sublethal to assess host morbidity) or 500 PFU/mouse (lethal to assess mortality) influenza A/PR8. IAV-infected mice were treated daily via intraperitoneal injection of either PBS or AST IV (20 mg/kg) from days 1 to 7 dpi. Body weight changes and survival rates were monitored daily.

### Quantitative RT-PCR (qRT‒PCR)

Briefly, RNA was extracted from cells using RNAiso Plus (TaKaRa, Beijing), and cDNA was synthesized via reverse transcription using 5 × RT Master Mix (TaKaRa, Beijing). qRT-PCR was performed with Fast SYBR Green PCR Master Mix (TaKaRa, Beijing). Gene expression data were analysed using the 2^−ΔΔCT^ method [20]. The sequences of primers used in this study are listed in Table 1.

**Table 1:** **qRT-PCR primers**

Gene	Primer sequences (5ʹ-3ʹ)
IL-1β	F: GGGCCTCAAAGGAAAGAATC R: TACCAGTTGGGGAACTCTGC
IL-6	F: GGTGGCCCAGTACCAATGC R: GGACCTGGACCACGTGCT
TNF-α	F: CATGCGTCCAGCTGACTAAA R: TCCCCTTCATCTTCCTCCTT
CCL2	F: GTCACCAAGCTCAAGAGAGAGGTC R: CCTACAGAAGTGCTTGAGGTGGTT
Sftpb	F: CTGTGCCAAGAGTGTGAGGA R: TTGGGGTTAATCTGGCTCTG
Abca3	F: TTCTGGTTCTCCGCTCTGTT R: GTACATGAGGGGGATGATGG
Wnt3a	F: GGGACCCCAGTACTCCTCTC R: GGGCATGATCTCCACGTAGT
FZD2	F: ACATCGCCTACAACCAGACC R: CGGGTAGAACTGATGCACCT
AXIN2	F: TAGGCGGAATGAAGATGGAC R: CTGGTCACCCAACAAGGAGT
CTNNB1	F: CAGTTTTGAACAAGTCGCTGAC R: GTGCTGAAGGTGCTGTCTG
NDUFS6	F: CCGACAGTGGTTGTGGATAATG R: GGTGTGTTTCCTGGTAGCATCA
NDUFA11	F: CTGTGTGGTGGAACCAAGAG R: GGGCCAGTGTAGGTAGTGGA
UQCR10	F: GGACCTGAACCCCAATGCTA R: GGTCGTTCCACACACACCTA
UQCR11	F: CCTGACCTCAGGTTGTTTCC R: GTCAGCCACACTGCTTTTGA
COX5A	F: GAGGACGACGTGATCAAGGA R: TCTTCCGAGCAGGACAAAGA
COX6A	F: TGAGCTGAGGAGCTGTTTGA R: GCATGTCCTCTTTCCACTCC
ATP5MF	F: GCTGGAGACCTTCAAGGACA R: CAGGAAGTCCATGTCCTTGC
TIMM17A	F: TGAGTGGAGGAGTCTGGAGT R: GGCAGGAGTGTGGAAAGGAT
HK2	F: CTTCTTCTGCCTTTGCCCAC R: GTTTCCTCTCCCTGACTGCC
ENO1	F: TGCAAGTTTGATCAGCCACT R: TGGAGGATGTTCTTGAGGCT
GLUT1	F: CGACAGCGACCTGGTGAAGA R: AGCCACGATGCTCAGATAGG
LDHA	F: TGGCTTTCTCCCTCACAGAC R: CCCAACCCCAACAACTTTCT
HPRT	F: CTCCGCCGGCTTCCTCCTCA R: ACCTGGTTCATCATCGCTAATC

### Virus titre measurement

To determine the viral titre, a 50% tissue culture infectious dose (TCID_50_) assay was performed using Madin–Darby canine kidney (MDCK) cells. The cells were seeded in 96-well plates at a density of 1 × 10^4^ cells/well and incubated overnight at 37 °C with 5% CO_2_ to reach 90–100% confluency. Serial tenfold dilutions of the BAL fluid were prepared in serum-free DMEM with 2 µg/mL TPCK-treated trypsin. Each dilution was added in quadruplicate to the MDCK cells and incubated at 37 °C with 5% CO_2_ for 72 h. Cytopathic effects (CPE) were observed under a microscope, and wells showing CPE were recorded for each dilution. The TCID_50_ was calculated using the Reed‒Muench method [21].

### Bronchoalveolar lavage (BAL) collection

BAL fluid was collected by flushing the airway with sterile PBS through a tracheal incision. The cells in the BAL fluid were pelleted by centrifugation, and the supernatants were collected for the analysis of cytokine and chemokine levels, protein concentrations, and viral titres.

### Enzyme-linked immunosorbent assay (ELISA)

A total of 600 μL of each BAL sample was analysed by ELISA to measure the protein levels of mouse IL-1b, TNF-a, IL-6, and CCL2 following the manufacturer’s protocol (Beyotime, Beijing, China). The results were read at a wavelength of 450 nm.

### Mouse AM culture and treatment in vitro

AM were isolated from BAL fluid obtained by flushing the airways. The collected fluid was centrifuged at 1500 rpm for 5 min at 4 °C to pellet the cells. The cell pellet was subsequently resuspended in RPMI 1640 medium supplemented with 10% FBS and 1% penicillin‒streptomycin. AM were purified by adhesion to tissue culture plates for 2 h at 37 °C in a 5% CO_2_ incubator. Nonadherent cells were removed by washing with PBS. AM were cultured in complete medium supplemented with recombinant murine GM-CSF (10 ng/mL) (MCE, NJ). For AST IV treatment, purified AM from wild-type C57BL/6 mice were incubated overnight in vitro with PBS or AST IV (20 μM, MCE) and further incubated with poly(I:C) (5 µg/mL, MCE). For Wnt activator treatment, AM were isolated from wild-type C57BL/6 mice and then incubated with AST IV overnight. Next, the Wnt activator (100 ng/mL, MCE) was further incubated with AM for 24 h [22]. The cells were subsequently harvested for further analysis.

### Network pharmacology analysis

The isomeric SMILES value and 3D structure of AST IV were downloaded from the PubChem database [23] and further imported into the SwissTargetPrediction [24], SuperPred [25], and CTD databases [26] to predict potential binding targets. All the predicted targets were subsequently integrated into the UniProt database [27] to systematically compile the target profile of AST IV. Moreover, influenza- and COVID-19-related targets were retrieved and collected from the GeneCards [28] and OMIM disease databases [29]. The potential targets of AST IV and the related targets of influenza and COVID-19 were intersected using the Venny 2.1.0 plotting tool [30]. This analysis identified the common targets among AST IV, influenza, and COVID-19, which were subsequently visualized in a Venn diagram.

The identified common targets were imported into the STRING database [31], “Multiple proteins” were selected, and the species was specified as *Homo sapiens*, with a combined score threshold of > 0.95. A protein‒protein interaction (PPI) network diagram was subsequently generated and downloaded in TSV format. The TSV file was then imported into Cytoscape 3.10.2, where the CytoNCA plugin was used to analyse and refine the PPI network diagram [32].

Enrichment analysis of the identified common targets was performed via the DAVID database, with the species set as *Homo sapiens* [33]. The analysis yielded results for Gene Ontology (GO) functions and Kyoto Encyclopedia of Genes and Genomes (KEGG) pathway enrichment, which were saved in an Excel file and systematically categorized [34, 35]. GO and KEGG enrichment results with a significance threshold of *P* < 0.05 were selected. The filtered results were then imported into Wei Sheng Xin for visualization [36]. Finally, GO function and KEGG pathway enrichment diagrams were generated and analysed, with statistical significance set at *P* < 0.05.

### Molecular docking

Molecular docking was performed to analyse the interaction between astragaloside IV and mouse β-catenin. The structure of astragaloside IV was optimized and saved in PDB format. The crystal structure of mouse β-catenin was retrieved from the Protein Data Bank and prepared [37]. Docking simulations were conducted via AutoDock Vina, with a grid box centered on the β-catenin active site [38]. The docking results were analysed via PyMOL to identify the binding affinity and key interactions between astragaloside IV and β-catenin residues [39].

### Metabolic analysis

The oxygen consumption rate (OCR) and extracellular acidification rate (ECAR) of AM were assessed in real time via a Seahorse XFp Analyser (Seahorse Bioscience) [40]. For the in vitro experiment, AM (1 × 10^5^ cells/well) isolated from wild-type mice were seeded into Seahorse XFp Cell Culture Miniplates and pretreated overnight at 37 °C with 5% CO_2_ either with PBS or AST IV (20 μM, MCE) and further incubated with a Wnt activator (100 ng/mL) or Poly(I:C) (5 µg/mL) for 24 h. For the in vivo experiment, AM were isolated from wild-type mice treated with PBS or AST IV (20 mg/kg) and further seeded into Seahorse XFp Cell Culture Miniplates and pretreated overnight at 37 °C with 5% CO_2_ for 24 h. Next, the cells were washed twice and incubated for 1 h at 37 °C in unbuffered assay medium (pH 7.4; Agilent Technologies) in a CO_2_-free environment. For the mitochondrial stress test, the medium contained 10 mM glucose, whereas for the glycolytic stress test, glucose was excluded. The OCR and ECAR were measured under basal conditions and after sequentially adding 1 mM oligomycin, 1.5 mM carbonyl cyanide-4-(trifluoromethoxy) phenylhydrazone (FCCP), 0.5 mM rotenone + 0.5 mM antimycin, 10 mM glucose, and 50 mM 2-deoxy-d-glucose (2-DG) (all drugs purchased from Sigma).

### Measurement of mitochondrial mass

AM **(**1 × 10^5)^ were plated in 24-well plates and treated with or without poly(I:C) (5 μg/mL) overnight at 37 °C in a 5% CO_2_ atmosphere. Then, the cells were washed and stained with MitoTracker Deep Red FM and MitoTracker Green (50 nM each; Beyotime) for 30 min at 37 °C. After staining, the cells were washed twice with PBS, detached from the wells, and subjected to flow cytometry analysis.

### Western blotting (WB)

The cells were lysed to extract total proteins, which were denatured in 1 × SDS loading buffer, separated by SDS‒PAGE, and transferred onto nitrocellulose (NC) membranes. The membranes were blocked with 5% skim milk for 1 h and incubated overnight with primary antibodies, including rabbit anti-active-β-catenin (CST, MA) or mouse anti-actin (Proteintech, Wuhan, China). Following incubation, the membranes were washed six times with Tris-buffered saline containing Tween-20 (TBST). The membranes were then incubated with horseradish peroxidase-conjugated secondary antibodies for 1 h and washed six more times with TBST [41]. The protein bands were detected using an enhanced chemiluminescence reagent (Thermo Fisher Scientific, IL, USA).

### Statistical analysis

The data are presented as the means ± SEM. Statistical significance was determined using unpaired two-tailed Student’s *t* test for two-group comparisons, one-way analysis of variance (ANOVA) for multiple-group comparisons, multiple t tests for weight loss analysis, or the log-rank test for survival studies. Analyses were performed using GraphPad Prism software. *P < 0.05 indicates a significant difference.

## Results

### AST IV targets alveolar macrophages to mitigate IAV-induced lung damage

**Figure 1:**
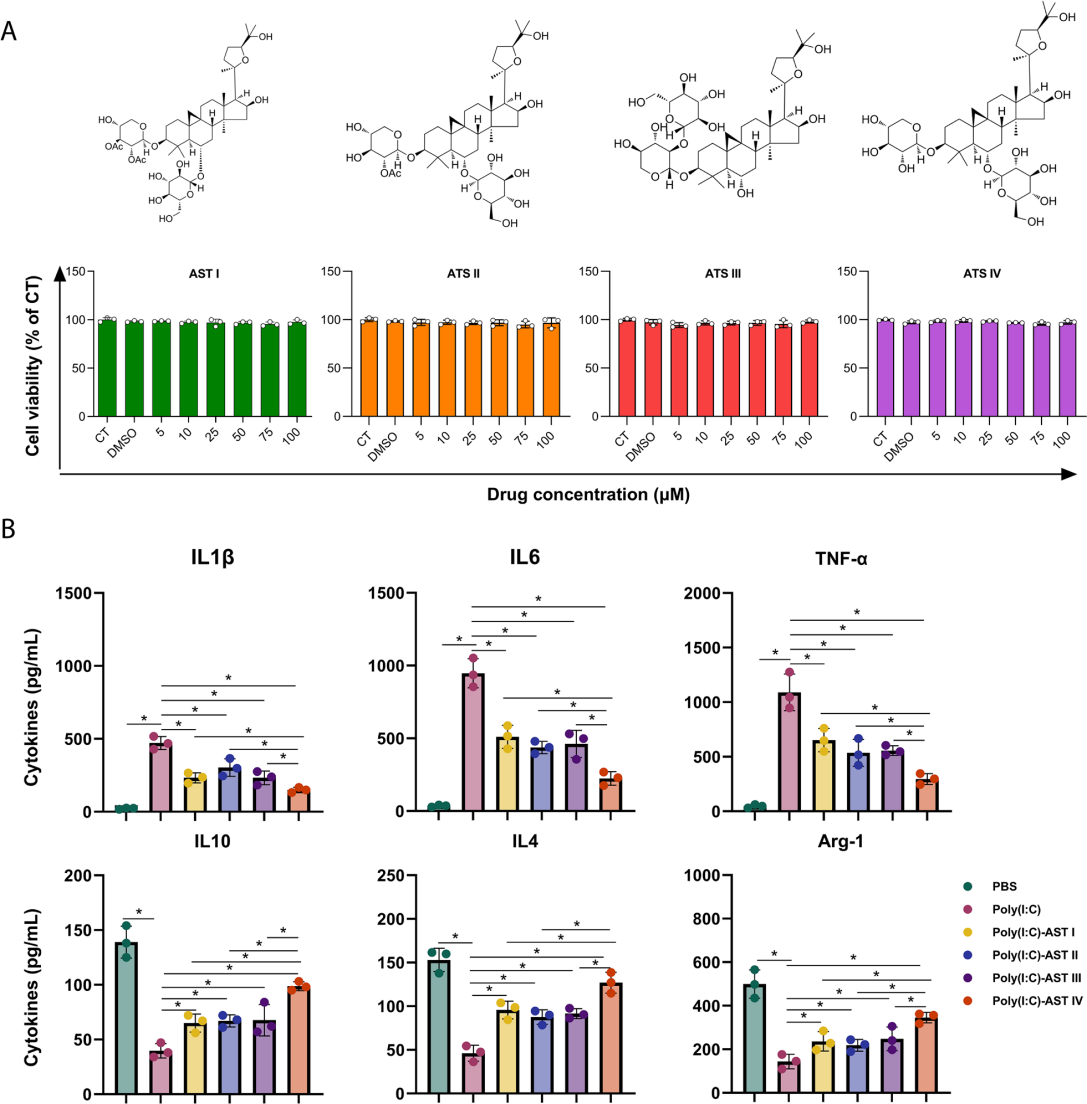
**Evaluation of the cytotoxicity and anti-inflammatory activity of astragalosides I-IV.** **A** MH-S cell viability was determined using the MTS assay after the cells were cultured with astragalosides I-IV at the specified concentrations. **B** MH-S cells were treated with astragalosides I-IV, and the levels of inflammatory cytokines (IL-1β, IL-6, and TNF-α) and anti-inflammatory cytokines (IL-4, IL-10, and Arg-1) were measured by ELISA. The data are presented as the means ± SEM. *P < 0.05.

**Figure 2:**
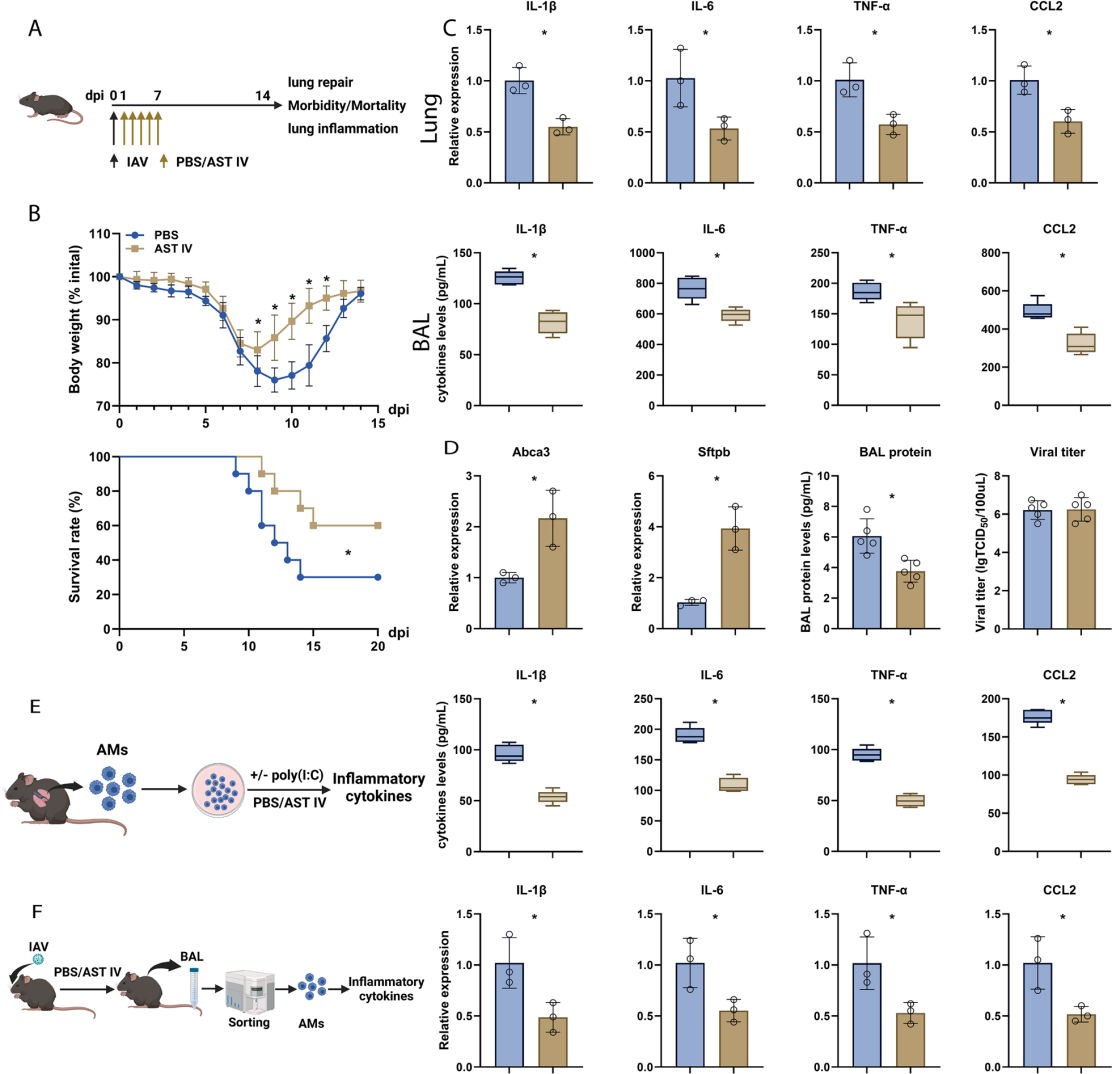
**AST IV targets alveolar macrophages (AM) to mitigate virus-mediated lung inflammation.** **A** Schematic diagram of the animal experiments (*n* = 12). **B** Mice treated with PBS or AST IV were infected with sublethal or lethal doses of IAV. Host morbidity and mortality were monitored. **C** The levels of inflammatory genes in the lungs and inflammatory cytokines in the BAL fluid were measured at 4 dpi. **D** BAL virus titres were measured at 4 dpi. Abca3 and Sftpb gene expression in the lungs at 14 dpi. Protein concentrations in the BAL fluid at 14 dpi. **E** Protein levels of IL-1β, IL-6, TNF-α, and CCL2 in AM stimulated with or without poly (I:C) in the presence of PBS or AST IV overnight in vitro. **F** Expression levels of IL-1β, IL-6, TNF-α, and CCL2 mRNAs in AM isolated from IAV-infected lungs at 4 dpi. The data are presented as the means ± SEM. *P < 0.05.

The astragaloside family consists of four members: astragaloside I, astragaloside II, astragaloside III, and astragaloside IV. Our results demonstrated that AST I-IV exhibited negligible cytotoxicity to MH-S cells at the tested concentrations (Figure 1A). Among them, AST IV displayed the most potent anti-inflammatory activity, as evidenced by the significant reduction in the levels of proinflammatory cytokines (IL-1β, IL-6, and TNF-α) and the increase in the levels of anti-inflammatory cytokines (IL-4, IL-10, and Arg-1) (Figure 1B). Next, the mice were intraperitoneally administered AST IV or PBS before infection with H1N1 influenza A virus (Figure 2A). AST IV treatment significantly reduced both host morbidity and mortality following infection (Figure 2B). Notably, AST IV markedly suppressed the expression of inflammatory genes in the lungs at 4 days post infection (dpi) (Figure 2C). Consistently, proinflammatory cytokine levels in bronchoalveolar lavage (BAL) fluid were dramatically reduced at 4 dpi (Figure 2C). Furthermore, total BAL protein levels were diminished in AST IV-treated mice at 14 dpi (Figure 2D), indicating that AST IV alleviates IAV-induced lung injury. However, compared with control mice, AST IV-treated mice presented similar viral titres in the lungs (Figure 2D). Following alveolar injury, alveolar type II (ATII) cells proliferate and differentiate into alveolar type I (AT1) cells to facilitate alveolar epithelium repair [42]. The expression levels of Abca3 and Sftpb, key markers of ATII cells, were significantly increased in AST IV-treated mice at 14 dpi (Figure 2D), suggesting that AST IV promotes lung tissue recovery. Given the critical role of alveolar macrophages in driving excessive inflammatory responses and contributing to respiratory viral pathogenesis, we isolated AM and found that AST IV treatment significantly reduced poly(I:C)-induced inflammatory cytokine expression, as demonstrated by qRT‒PCR analysis (Figure 2E). Furthermore, AM were isolated from mice subjected to AST IV or PBS before IAV infection. Our data confirmed that AST IV significantly reduced inflammatory gene expression in AM (Figure 2F). Collectively, these findings suggest that AST IV targets alveolar macrophages to protect against respiratory viral infection.

### AST IV promotes mitochondrial fitness in AM

**Figure 3:**
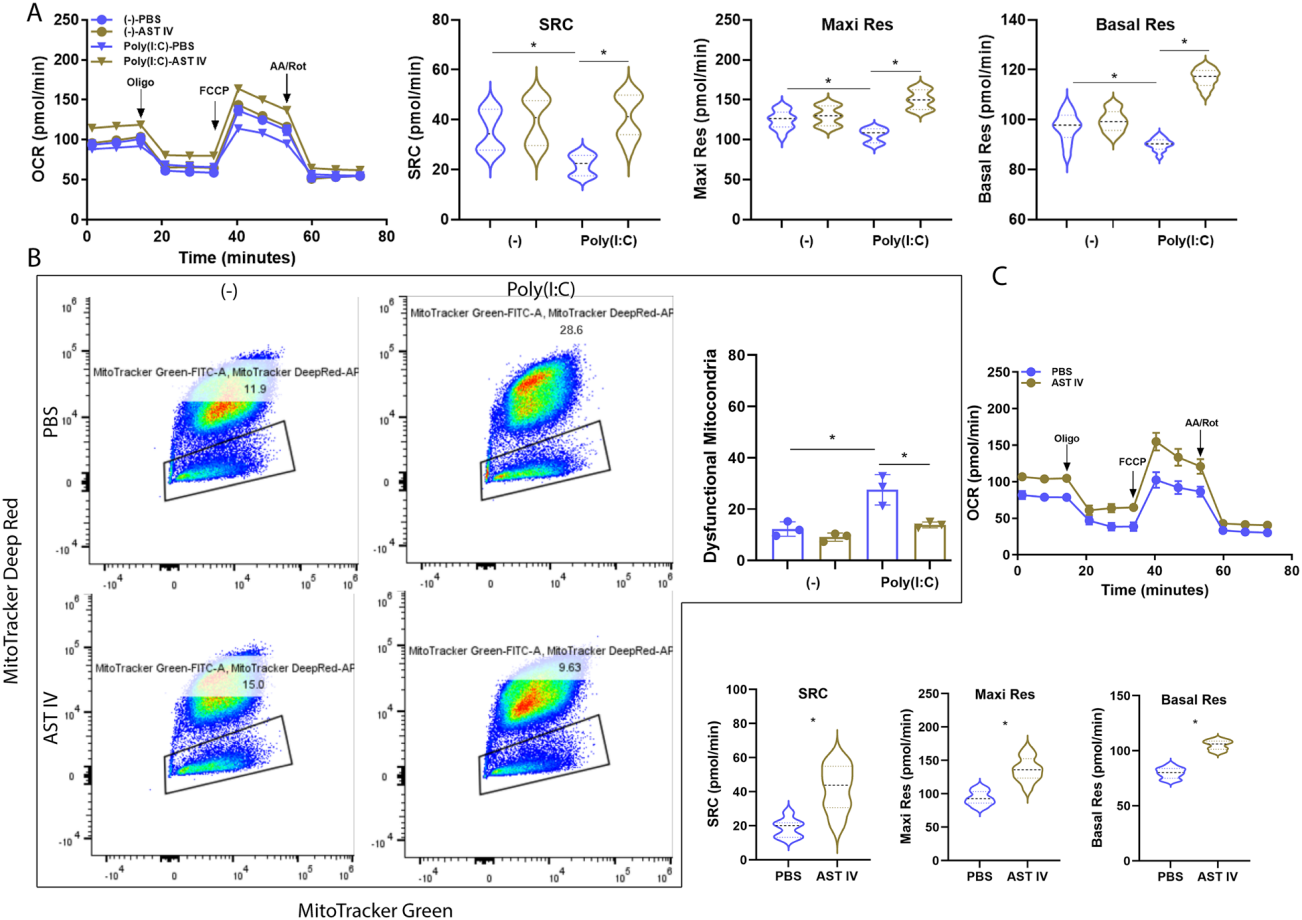
**AST IV promotes mitochondrial fitness in AM.** **A**, **B** AM were incubated overnight in vitro with or without poly(I:C) in the presence of PBS or AST IV. **A** AM were treated with oligomycin (Oligo), carbonyl cyanide-4-(trifluoromethoxy) phenylhydrazone (FCCP), and antimycin A/rotenone (AA/Rot) to measure the oxygen consumption rate (OCR). The spare respiratory capacity (SRC), maximal respiratory capacity (Maxi Res), and basal respiration (Basal Res) were quantified. **B** Flow cytometry analysis was performed to assess the mitochondrial mass using Mito Tracker Green and MitoTracker Deep Red in AM. **C** OCR of AM from naïve or IAV-infected WT mice treated with PBS or AST IV at 4 dpi. (*n* = 5). SRC, Maxi Res, and Basal Res were quantified. The data are presented as the means ± SEM. *P < 0.05.

**Figure 4:**
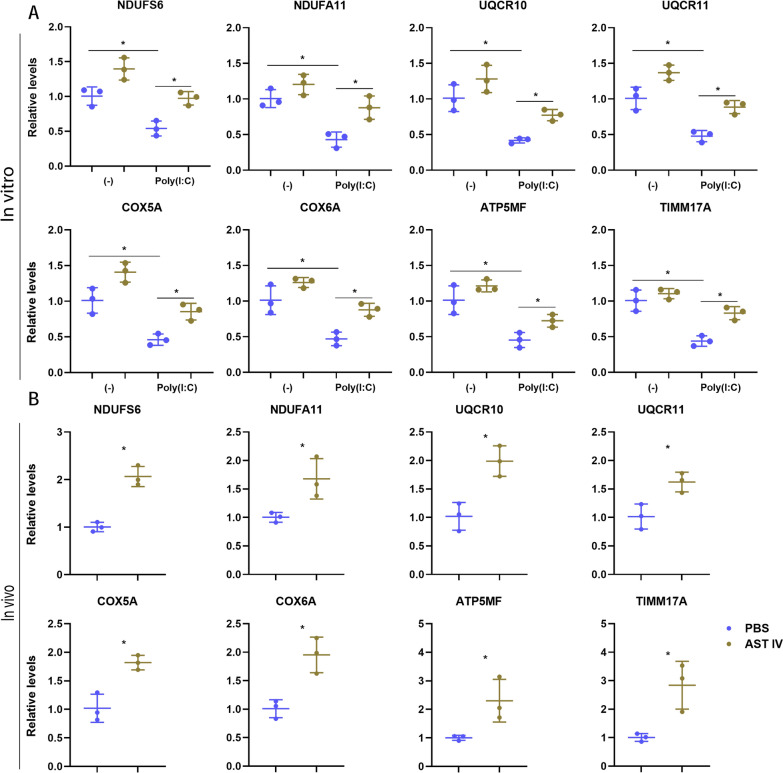
**AST IV contributes to mitochondrial related genes expression in AM.** **A**, **B** RT-qPCR was conducted to assess the expression levels of genes related to mitochondrial OXPHOS complexes in AM incubated overnight in vitro with or without Poly(I:C) in the presence of PBS or AST IV. **A** Consistently, AM from naïve or IAV-infected WT mice treated with PBS or AST IV at 4 dpi were subjected to qPCR (**B**). Additionally, AM were incubated overnight in vitro with or without poly(I:C) in the presence of PBS or AST IV before analysis. The data are presented as the means ± SEM. *P < 0.05.

Mitochondria play a crucial role in regulating the innate immune response of host cells to viral infections [43]. In the context of alveolar macrophages, viral infection suppresses mitochondrial metabolism, thereby amplifying macrophage-mediated inflammatory responses [44]. Here, we assessed mitochondrial fitness by detecting the oxygen consumption rate (OCR). Our results revealed that Poly(I:C) treatment significantly reduced the spare respiratory capacity (SRC), maximal respiratory capacity (Maxi Res), and basal respiration (Basal Res) in AM compared with those in the control group. However, AST IV treatment effectively abrogated the reduction in SRC, Maxi Res, and Basal Res induced by Poly(I:C) (Figure 3A). Next, we determined the health state of the mitochondria. Consistently, depolarized mitochondria were significantly increased after Poly(I:C) treatment, whereas AST IV mitigated the mitochondrial depolarization caused by Poly(I:C) treatment (Figure 3B). Furthermore, AM were isolated from IAV-infected mice treated with AST IV or PBS and subjected to OCR detection. Our results revealed that AST IV treatment greatly increased the SRC, Maxi Res, and Basal Res levels during IAV infection (Figure 3C). We further performed RT-qPCR to determine whether AST IV enhances the expression levels of mitochondrial OXPHOS complex-related genes during IAV infection (Figure 4B). AST IV treatment dramatically increased the expression of the complex I subunits NADH ubiquinone oxidoreductase subunits S6 and A11 (NDUFS6, NDUFA11); complex III subunits-ubiquinol-cytochrome C reductase subunit X, XI (UQCR10, UQCR11); cytochrome C oxidase complex subunits 5A and 6A (COX5A, COX6A); ATP synthase membrane subunit F (ATP5MF); and translocase of the inner mitochondrial membrane (TIMM17A). Consistently, AST IV significantly abrogated the Poly(I:C)-induced downregulation of NDUFS6, NDUFA11, UQCR10, UQCR11, COX5A, COX6A, ATP5MF, and TIMM17A (Figure 4A). Therefore, our data indicate that AST IV maintains mitochondrial fitness in AM.

### AST IV mitigates the inflammatory response by suppressing the Wnt/β-catenin signalling pathway

**Figure 5:**
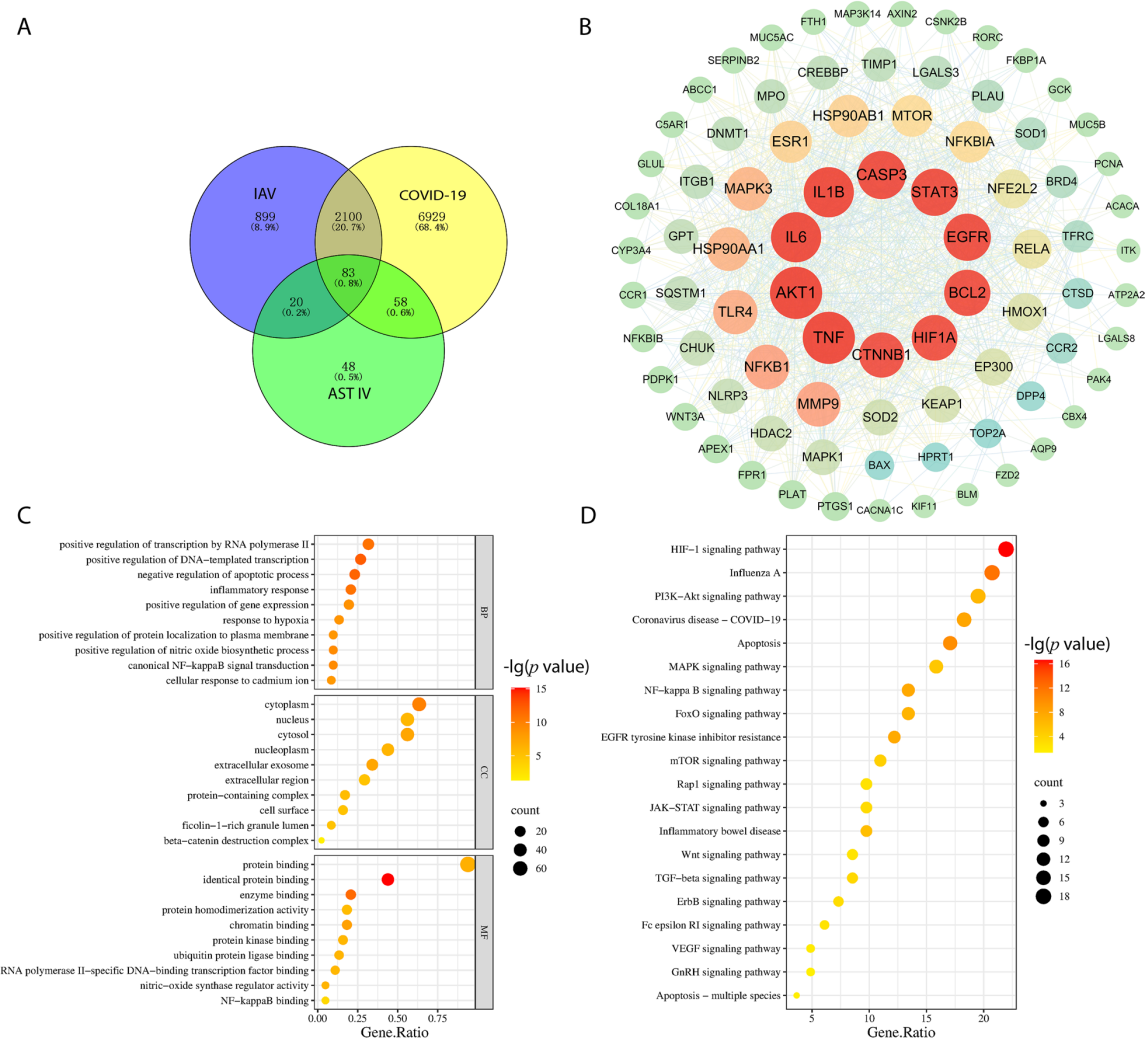
**Network pharmacology analysis of astragalosides IV, IAV, and COVID-19.** **A** Overlap of potential astragaloside IV targets and IAV- or COVID-19-related targets. **B** The protein‒protein interaction (PPI) network of the astragalosides IV-IAV/COVID-19 crossover genes. **C**, **D** Enrichment analysis and gene‒pathway network analysis. **C** GO enrichment analysis of core targets of astragaloside IV in the treatment of IAV/COVID-19. **D** Bubble chart of KEGG pathway annotation.

**Figure 6:**
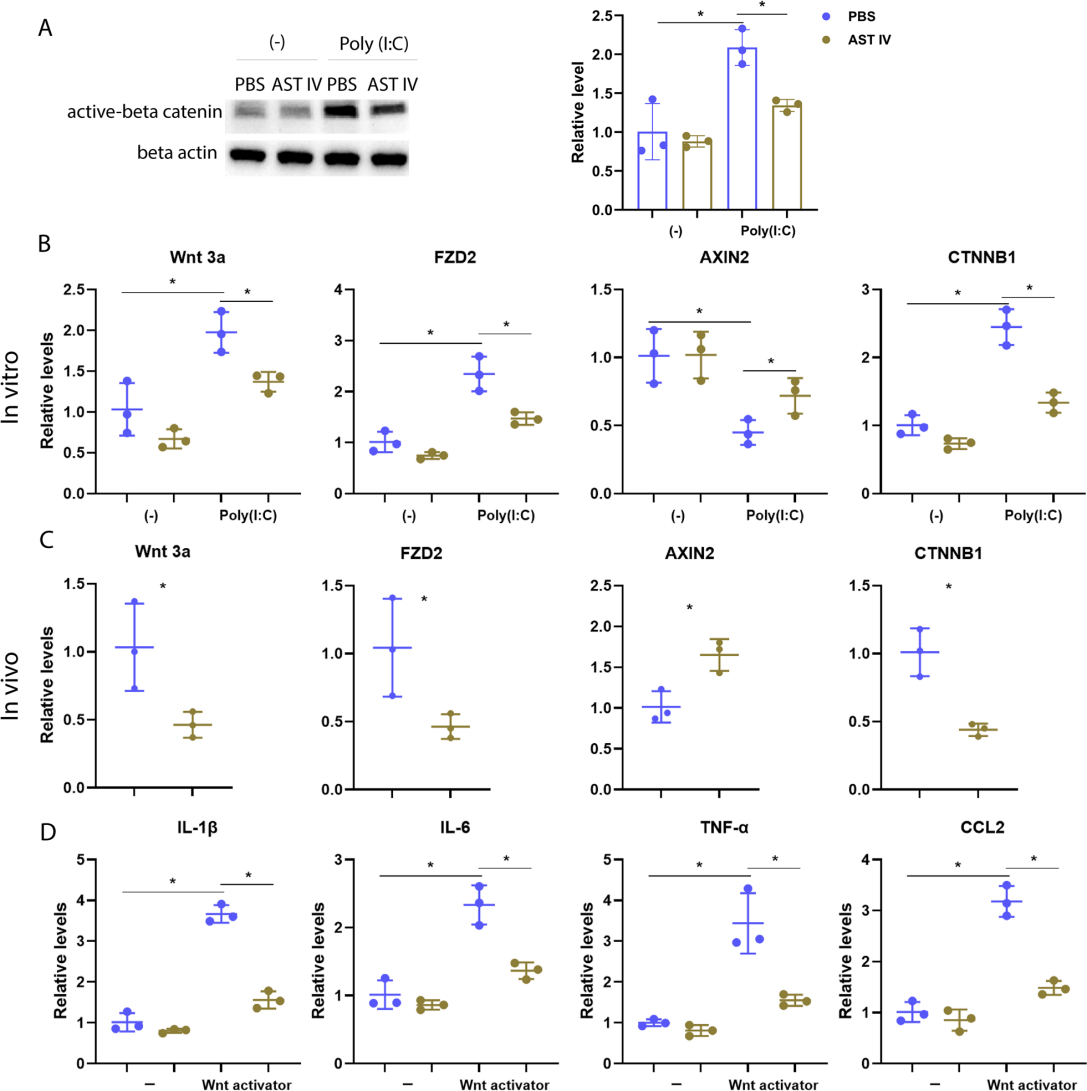
**AST IV mitigates the inflammatory response by suppressing the Wnt/β-catenin signalling pathway.** **A** Protein levels of active β-catenin in AM stimulated with or without poly(I:C) in the presence of PBS or AST IV were determined by western blotting. **B**, **C** Gene expression levels of Wnt3a, FZD2, AXIN2, and CTNNB1 in AM stimulated with or without Poly(I:C) in the presence of PBS or AST IV overnight in vitro. For the in vivo experiment, the expression levels of Wnt3a, FZD2, AXIN2, and CTNNB1 in AM from naïve or IAV-infected WT mice treated with PBS or AST IV at 4 dpi were measured (**C**). **D** Gene expression levels of IL-1β, IL-6, TNF-α, and CCL2 in AM stimulated with or without the Wnt activator in the presence of PBS or AST IV overnight in vitro.

AST IV plays a dual role in regulating the Wnt/β‑catenin signalling pathway [18, 19]. Notably, Wnt/β‑catenin drives the inflammatory response in AM [17]. Hence, the effect of AST IV on the Wnt/β‑catenin signalling pathway in AM was further investigated. First, we utilized network pharmacology to identify the core common targets of AST IV, IAV, and COVID-19. As shown in our results (Figure 5A and B), 83 common targets were identified, with CTNNB1, the gene encoding β-catenin, being among the top 10 genes. Next, GO functional enrichment analysis was conducted, which included biological processes, cellular components, and molecular functions. The results revealed that the target genes were associated primarily with apoptosis, inflammation, and hypoxia. Notably, for cellular components, the β-catenin destruction complex was enriched (Figure 5C). Consistently, KEGG pathway analysis revealed enrichment of pathways related to apoptosis, inflammatory signalling, HIF-1α signalling, and Wnt signalling (Figure 5D). Together, these findings suggest that AST IV may regulate the Wnt/β‑catenin signalling pathway during respiratory viral infections. To investigate whether AST IV binds to β-catenin, molecular docking analysis was performed. AST IV demonstrated a high affinity for β-catenin, with an affinity score of − 8.4 (Additional file 1). To confirm our hypothesis, AM were isolated and incubated with AST IV. We found that AST IV treatment greatly decreased the protein level of active β‑catenin in the context of poly(I:C) treatment (Figure 6A). To further determine the inhibition of the Wnt/β‑catenin signalling pathway. We detected the expression levels of key signalling genes, including Wnt3a, FZD2, CTNNB1, and AXIN2. The qRT-PCR results revealed that AST IV treatment significantly reduced the levels of Wnt3a, FZD2, and CTNNB1 while increasing AXIN2 gene expression (Figure 6B). Furthermore, AM were isolated from IAV-infected mice treated with AST IV or PBS. Consistently, AST IV treatment led to a reduction in Wnt3a, FZD2, and CTNNB1 expression, which was accompanied by the upregulation of AXIN2. (Figure 6C). These data indicate that AST IV suppressed the Wnt/β‑catenin signalling pathway in AM. Furthermore, we isolated and treated AM with a Wnt activator, followed by AST IV treatment. The inflammatory cytokine gene expression levels were assessed via qRT‒PCR. We found that the Wnt activator increased the gene expression of IL-1β, IL-6, TNF-α, and CCL2, whereas further AST IV treatment significantly decreased the IL-1β, IL-6, TNF-α, and CCL2 levels (Figure 6D). Thus, AST IV mitigates inflammation through antagonizing the Wnt/β‑catenin signalling pathway in AM.

### AST IV suppresses glycolysis in AM both in vivo and in vitro

**Figure 7:**
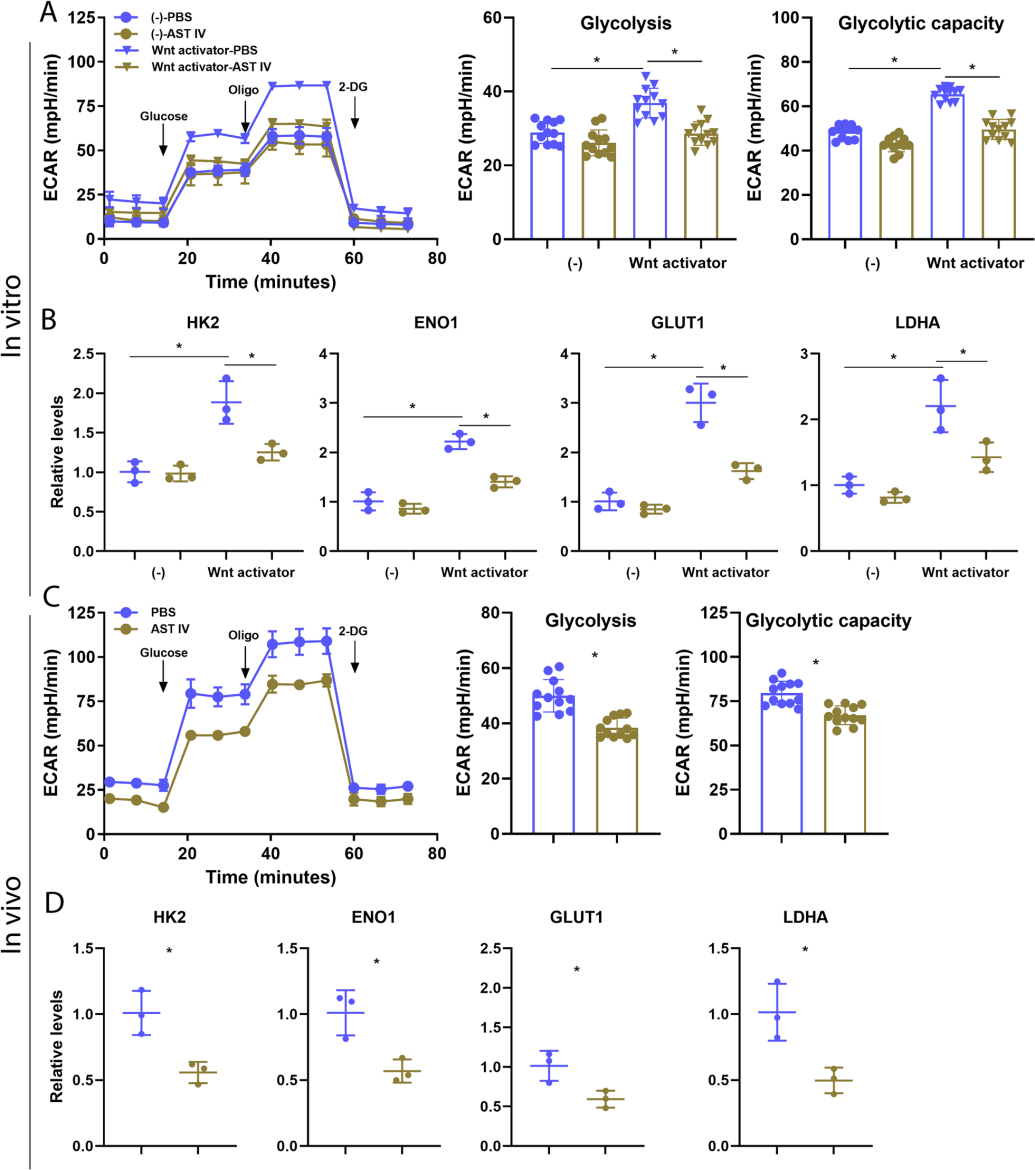
**AST IV alleviates glycolysis in AM.** **A** AM were incubated overnight in vitro with or without a Wnt activator in the presence of PBS or AST IV. The extracellular acidification rate (ECAR) was measured in real time through the sequential addition of glucose, oligomycin, and 2-deoxyglucose (2-DG). Glycolysis and the glycolytic capacity in AM were subsequently evaluated. **B** The expression levels of key glycolysis-related genes were measured by qRT-PCR. **C** In the in vivo experiment, glycolysis and glycolytic capacity were evaluated in AM from naïve or IAV-infected WT mice treated with PBS or AST IV at 4 dpi. **D** The expression levels of key glycolysis-related genes were measured by qRT‒PCR. The data are presented as the means ± SEM. *P < 0.05.

The Wnt/β‑catenin signalling pathway drives glycolysis to trigger inflammation in AM [17]. Therefore, we assessed changes in glycolytic ability by detecting the extracellular acidification rate (ECAR). Wnt activator treatment increased the extracellular acidification rate, as evidenced by the increased glycolysis level and glycolytic capacity, whereas AST IV treatment dramatically decreased the level of glycolysis and glycolytic capacity (Figure 7A). Moreover, we isolated AM from IAV-infected mice treated with PBS or AST IV. Consistently, AST IV significantly reduced the level of glycolysis and glycolytic capacity induced by IAV infection (Figure 7C). To confirm our results, we further investigated alterations in the glycolysis marker genes *HK2*, *ENO1*, *GLUT1*, and *LDHA*. As shown by our results, Wnt activator treatment significantly increased the expression of core glycolytic genes, whereas AST IV treatment dramatically mitigated the increased expression levels of these genes (Figure 7B). Consistently, AST IV treatment significantly decreased glycolytic gene expression during IAV infection (Figure 7D). Overall, our results showed that AST IV treatment dampens glycolysis by suppressing the Wnt/β‑catenin signalling pathway.

## Discussion

During respiratory virus infection, inflammation is elicited to defend against pathogen invasion. However, excessive inflammatory responses can cause tissue damage and severe disease [5, 6]. AM are key components of the innate immune system and provide a first line of defense against respiratory viruses [5]. AM maintain pulmonary homeostasis by removing pathogens and cellular debris through phagocytosis and secreting various cytokines to regulate immune responses. However, the underlying mechanism by which inflammation is balanced in AM requires further investigation. Here, we explored the protective effects of AST IV against IAV infection. Our findings demonstrate that AST IV significantly reduces morbidity and mortality in mice infected with IAV. This protective effect is accompanied by increased lung recovery capacity. Importantly, we revealed that AST IV exerts its protective effects by targeting AM and suppressing the Wnt/β-catenin signalling pathway, a key regulator of inflammatory responses. Overall, our study highlights AST IV as a promising therapeutic candidate for managing respiratory viral infections.

AST IV, a bioactive compound derived from the traditional medicinal herb *Astragalus membranaceus*, has been shown to possess potent immunomodulatory properties. Previous studies have demonstrated that AST IV regulates innate immune responses by modulating various signalling pathways, including those involved in inflammation [7–9]. For example, AST IV has been shown to modulate microglial polarization through the PI3K/Akt signalling pathway, thereby alleviating neuroinflammation and brain injury [7]. However, an increasing number of studies have indicated that AST IV mitigates lung inflammation induced by silica, PM 2.5, LPS, and bleomycin [45–48]. Notably, previous studies have suggested that AST IV mitigates IAV-induced inflammation through enhancing autophagy while suppressing the NLRP3/Caspase-1 signalling pathway [10, 11]. Owing to the complex mechanisms underlying the inflammation induced by the influenza virus, the specific mechanism of the anti-inflammatory effects of AST IV still requires further investigation. Our study demonstrated that AST IV significantly suppressed proinflammatory cytokine levels in the lungs and AM of IAV-infected mice, thereby mitigating lung damage (Figure 2). Moreover, AST IV facilitates lung recovery by enhancing the reparative capacity of AT II cells. As the primary defense against respiratory viruses, AM activation leads to cytokine production and inflammation, which subsequently recruit other immune cells to facilitate pathogen clearance. Furthermore, AM can secrete several factors (transforming growth factor-β, keratinocyte growth factor) to support alveolar epithelial repair and proliferation [49, 50]. Therefore, although AST IV promotes AT II epithelial cell regeneration, we are more concerned about its effects on AM. Specifically, we aimed to determine whether AST IV directly targets AM to mitigate inflammation induced by IAV. Next, we isolated these cells and conducted both in vitro and in vivo drug treatment experiments. AST IV effectively inhibited the production of inflammatory cytokines triggered by poly (I:C) treatment or IAV infection. These findings suggest that AST IV primarily modulates lung inflammation by targeting AM.

The Wnt/β-catenin signalling pathway is a central player in regulating numerous cellular processes, including proliferation, differentiation, and tissue homeostasis [12, 13]. Recent research has highlighted the dual role of β-catenin in regulating inflammation. On the one hand, β-catenin can suppress proinflammatory signalling; on the other hand, it can promote inflammatory responses by modulating the activity of key transcription factors such as NF-κB [51, 52]. This context-dependent regulation of inflammation makes Wnt/β-catenin signalling a promising target for therapeutic intervention, especially in diseases involving excessive or chronic inflammation, such as respiratory viral infections. In AM, Wnt/β-catenin signalling drives inflammation and contributes to lung damage during respiratory viral infections [17]. Interestingly, AST IV has been shown to exert opposing effects on Wnt/β-catenin signalling in different contexts [18, 19]. Therefore, further investigations are needed to determine whether AST IV regulates inflammation in AM through the Wnt/β-catenin signalling pathway. Our network pharmacology analysis revealed that CTNNB1 was among the top 10 common targets shared by AST IV, IAV, and COVID-19 (Figure 5A–D). Furthermore, molecular docking data also support the link between AST IV and the Wnt/β-catenin pathway (Additional file 1). Specifically, AST IV treatment inhibited the activation of β-catenin in AM, leading to reduced inflammatory cytokine production (Figure 6). This finding is consistent with previous studies showing that β-catenin promotes inflammatory responses in macrophages and other immune cells, thereby exacerbating tissue damage during viral infections [17]. Thus, by targeting Wnt/β-catenin signalling, AST IV effectively attenuates the inflammatory response in AM, reducing lung injury and promoting recovery. Thus, by targeting the Wnt/β-catenin signalling pathway, AST IV effectively attenuates inflammatory responses in AM, thereby reducing lung injury and facilitating recovery.

Mitochondrial fitness and glycolysis are intimately linked to the inflammatory response in immune cells [53, 54]. In the context of respiratory viral infections, mitochondrial dysfunction and enhanced glycolysis contribute to the hyperactivation of macrophages, exacerbating the inflammatory response [55, 56]. Moreover, a previous study revealed that Wnt/β-catenin signalling promotes glycolysis while impairing mitochondrial fitness [17]. Our study demonstrated that AST IV promotes mitochondrial fitness in AM during IAV infection, restoring mitochondrial function. This effect was evident from the increased OCR and reduced mitochondrial depolarization in AST IV-treated AM both in vivo and in vitro (Figures 3 and 4). By maintaining mitochondrial fitness, AST IV may prevent the metabolic shift towards glycolysis, thereby dampening the inflammatory response. Our findings further support this link, showing that activation of Wnt signalling increases glycolysis in AM, whereas AST IV treatment suppresses this effect, reducing glycolytic gene expression and glycolytic capacity (Figure 7). The ability of AST IV to regulate inflammation underscores its potential as a therapeutic agent for respiratory viral infections.

**Figure 8:**
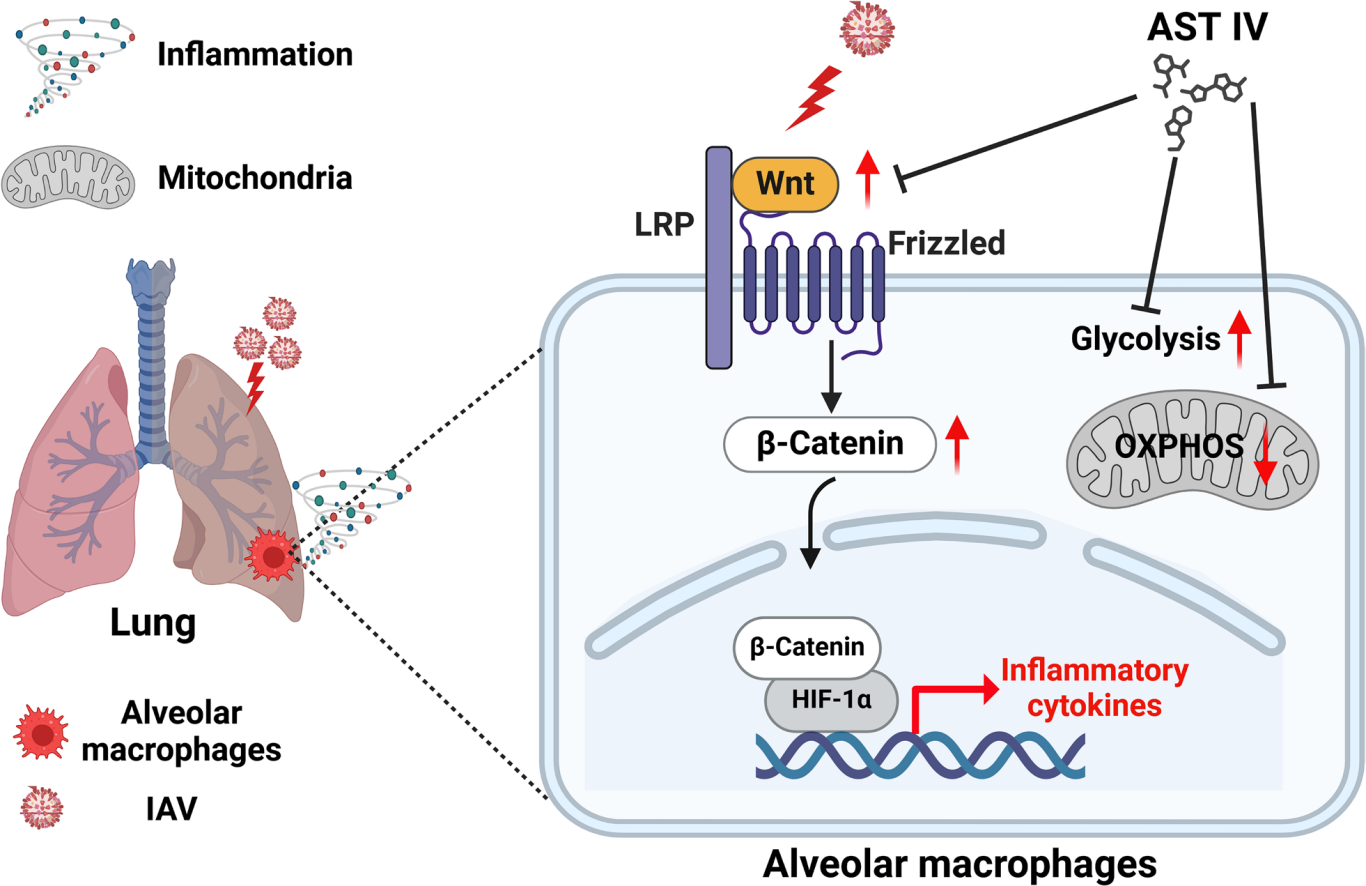
**Working model of AST IV.** AST IV mitigates IAV-induced lung inflammation by suppressing the Wnt/β-catenin signalling pathway, reducing glycolysis, and preserving mitochondrial fitness in AM.

In conclusion, our combined in vivo and in vitro experiments revealed that AST IV effectively modulates inflammation in AM during IAV infection by suppressing the Wnt/β-catenin signalling pathway, inhibiting glycolysis, and maintaining mitochondrial fitness (Figure 8). These actions collectively lead to reduced lung injury, enhanced tissue recovery, and improved survival outcomes in IAV-infected mice.

## Supplementary Information

**Additional file 1. Astragaloside IV exhibits high affinity for β-catenin. **(A) Molecular docking of Astragaloside IV with β-catenin. (B) Identification of key binding sites between Astragaloside IV and β-catenin.

## Data Availability

All of the data analysed in this work are included in this published article. The raw data generated in this study are available upon reasonable request.
