# Supplementary material for: Astragaloside IV mitigates influenza-induced inflammatory responses by suppressing the Wnt/β-catenin signalling pathway in alveolar macrophages
Source: Vet Res. 2025 Apr 30;56:95. doi: 10.1186/s13567-025-01529-5 (PMC12042467; doi:10.1186/s13567-025-01529-5)
Supplement: Supplementary file 1 — Additional file 1. Astragaloside IV exhibits high affinity for β-catenin. (A) Molecular docking of Astragaloside IV with β-catenin. (B) Identification of key binding sites between Astragaloside IV and β-catenin. [file 13567_2025_1529_MOESM1_ESM.pdf]

A

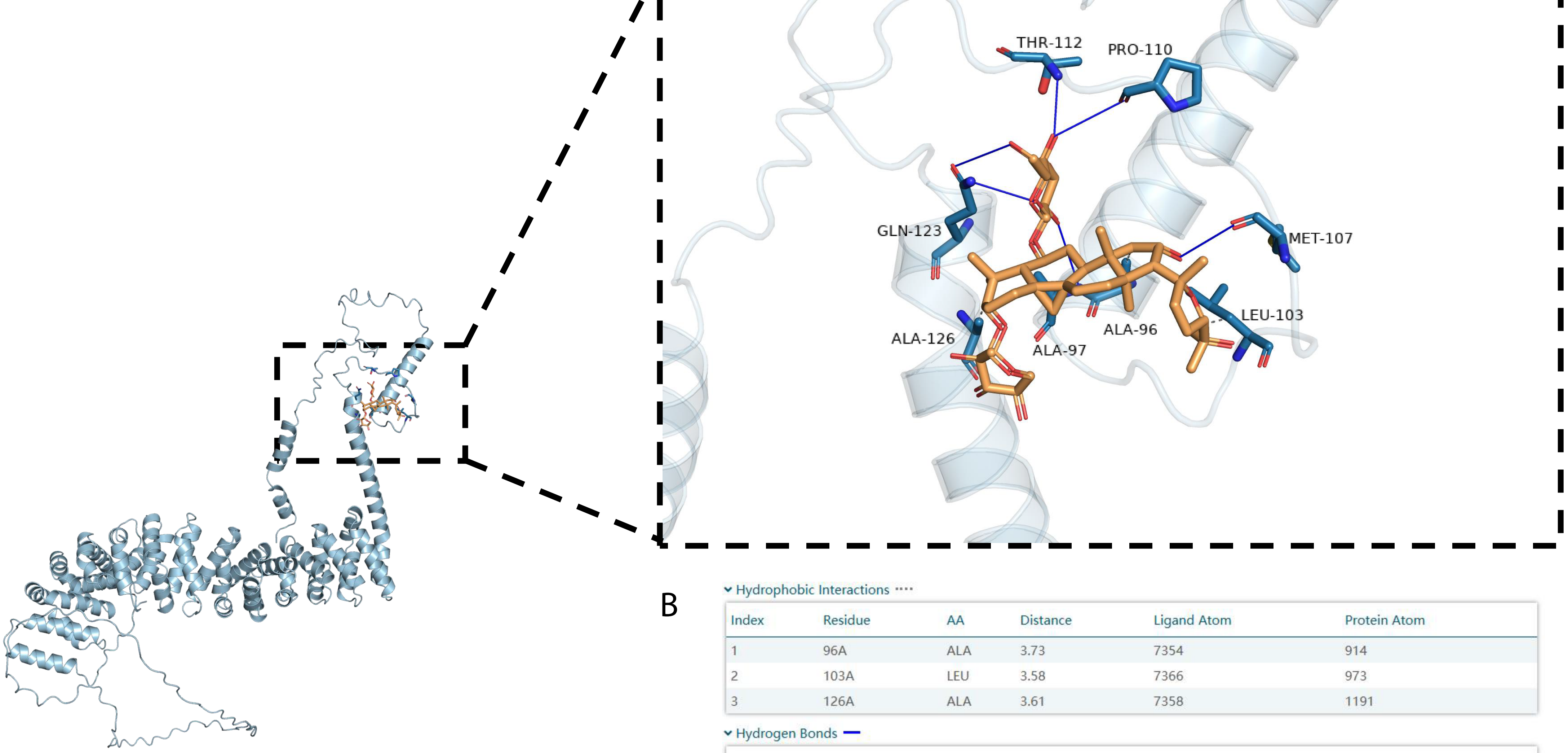


B

Hydrophobic Interactions ····

| Index | Residue | AA | Distance | Ligand Atom | Protein Atom |
|---|---|---|---|---|---|
| 1 | 96A | ALA | 3.73 | 7354 | 914 |
| 2 | 103A | LEU | 3.58 | 7366 | 973 |
| 3 | 126A | ALA | 3.61 | 7358 | 1191 |

Hydrogen Bonds —

| Index | Residue | AA | Distance H-A | Distance D-A | Donor Angle | Protein donor? | Side chain | Donor Atom | Acceptor Atom |
|---|---|---|---|---|---|---|---|---|---|
| 1 | 97A | ALA | 3.41 | 3.77 | 102.59 | ✔ | × | 916 [Nam] | 7386 [O3] |
| 2 | 107A | MET | 2.51 | 3.19 | 125.22 | × | × | 7381 [O3] | 1005 [O2] |
| 3 | 110A | PRO | 3.23 | 4.00 | 135.24 | × | × | 7384 [O3] | 1035 [O2] |
| 4 | 112A | THR | 2.34 | 3.21 | 141.63 | ✔ | × | 1047 [Nam] | 7384 [O3] |
| 5 | 123A | GLN | 2.72 | 3.27 | 114.14 | ✔ | ✔ | 1156 [Nam] | 7383 [O3] |
| 6 | 123A | GLN | 3.25 | 3.94 | 128.55 | × | ✔ | 7387 [O3] | 1157 [O2] |
